# Urine TWEAK level as a biomarker for early response to treatment in active lupus nephritis: a prospective multicentre study

**DOI:** 10.1136/lupus-2018-000298

**Published:** 2019-04-09

**Authors:** Thitima Benjachat Suttichet, Wonngarm Kittanamongkolchai, Chutipha Phromjeen, Sirirat Anutrakulchai, Thanachai Panaput, Atiporn Ingsathit, Nanticha Kamanamool, Vuddhidej Ophascharoensuk, Vasant Sumethakul, Yingyos Avihingsanon

**Affiliations:** 1 Department of Medicine, Faculty of Medicine, Center of Excellence in Immunology and Immune-mediated Diseases, Chulalongkorn University, Bangkok, Thailand; 2 Chula Clinical Research Center and Renal Immunology and Transplantation Research Unit, Faculty of Medicine, Chulalongkorn University, Bangkok, Thailand; 3 Division of Nephrology, Department of Medicine, Faculty of Medicine, Chulalongkorn University, Bangkok, Thailand; 4 Department of Medicine, Khon Kaen University, Khon Kaen, Thailand; 5 Department of Medicine, Khon Kaen Regional Hospital, Khon Kaen, Thailand; 6 Section for Clinical Epidemiology and Biostatistics, Faculty of Medicine, Ramathibodi Hospital, Mahidol University, Bangkok, Thailand; 7 Department of Preventive and Social Medicine, Faculty of Medicine, Srinakharinwirot University, Bangkok, Thailand; 8 Department of Internal Medicine, Faculty of Medicine, Chiang Mai University, Chiang Mai, Thailand; 9 Department of Internal Medicine, Faculty of Medicine, Ramathibodi Hospital, Mahidol University, Bangkok, Thailand

**Keywords:** biomarker, lupus nephritis, TWEAK, urine protein, response prediction

## Abstract

**Background:**

TNF-like weak inducer of apoptosis (TWEAK) is a proinflammatory molecule that plays a key role in active inflammation of lupus nephritis (LN). Urine TWEAK (uTWEAK) levels were found to be associated with renal disease activity among patients with LN. Here, we determined whether serial measurements of uTWEAK during induction therapy could predict treatment response or not.

**Methods:**

Spot urine samples were collected from patients with biopsy-proven active LN at time of flare, and 3 and 6 months after flare to assess the uTWEAK levels. All patients received standard immunosuppressive therapy and treatment response was evaluated at 6 months. The performance of uTWEAK as a predictor for treatment response was compared with clinically used biomarkers for patients with LN.

**Results:**

Among 110 patients with LN, there were 29% complete responders (CR), 34% partial responders (PR) and 37% non-responders (NR). On average, uTWEAK level was consistently low in CR, trended down by 3 months in PR and persistently elevated in NR. uTWEAK levels at month 3 were able to predict complete response at month 6 (OR adjusted for age, sex and creatinine=0.34 [95% CI 0.15 to 0.80], the area under the receiver operating characteristic curve [ROC-AUC]=0.68, p=0.02). The optimal threshold for uTWEAK level at month 3 was 0.46 pg/mgCr, discriminating complete response with 70% sensitivity and 63% specificity. Combining uTWEAK and urine protein at month 3 improved predictive performance for complete response at 6 months (ROC-AUC 0.83, p<0.001).

**Conclusions:**

In addition to urine protein, uTWEAK level at 3 months after flare can improve the accuracy in predicting complete response at 6 months of induction therapy.

## Background

Lupus nephritis (LN) is a major burden of SLE leading to end-stage renal disease. Despite aggressive immunosuppressive therapy, complete response rate remains unsatisfactory.^1 2^ One of the strategies to improve the outcome of LN and reduce treatment-related toxicity is to serially evaluate renal disease activity following initial therapy that would allow early optimisation of immunosuppression.^3^ Unfortunately, current biomarkers (complements and anti-dsDNA) are not sensitive nor specific.^4 5^ It also has been shown that almost half of the patients with LN who clinically respond to treatment had active lesions on repeat biopsies.^6^ Although spot urine protein has been proposed to be the best predictor of long-term kidney function,^7^ proteinuria can come from scarring or pure membranous lesions. Thus, the use of proteinuria as one of the criteria for treatment response of LN is not perfect. Renal biopsy remains to be the gold standard but is too dangerous to be repeated several times. Identification of novel biomarkers that have therapeutic guidance or prognostic significance is much needed. Urine biomarkers appear to be more attractive than serum biomarkers because they are easily obtainable and possibly the direct products of kidney inflammation or injury.^8^

TNF-like weak inducer of apoptosis (TWEAK) is a proinflammatory cytokine from the TNF superfamily that binds monogamously to its receptor Fn14.^9^ TWEAK plays a prominent role in the pathogenesis of LN through several intracellular signal transduction cascades and induces apoptosis of glomerular mesangial cells and tubular epithelial cells.^10^ In a mouse model of SLE, Fn14 deficiency or treatment with an anti-TWEAK antibody significantly reduced renal inflammation as well as proteinuria.^11^

Recently there has been a surge of interest in urine TWEAK (uTWEAK) as an LN biomarker to evaluate disease activity. Multiple prior studies have supported uTWEAK to be a candidate clinical biomarker for LN.^12–16^ uTWEAK levels were significantly higher in patients with SLE with active LN than those without, and also correlated with renal disease activity in patients who have been longitudinally followed.^12 13^ However, many of these studies were limited to cross-sectional design and had small sample size. In addition, the role of uTWEAK as a prognostic marker has not yet been examined. This study evaluated the value of uTWEAK in predicting the renal response to induction therapy.

## Methods

### Study participants

The study was based on three cohorts of patients: (1) the Chulalongkorn University Hospital (CU) cohort, including patients regularly followed in a specialised LN clinic in King Chulalongkorn Memorial Hospital from 2005 to 2012 (n=29); (2) the CONTROL study, a multicentre randomised controlled study to compare enteric-coated mycophenolate sodium (EC-MPS) and intravenous cyclophosphamide as an induction therapy for LN (n=42)^17^; and (3) the Thai Tacrolimus Trial (TTT), a multicentre, randomised controlled study to compare tacrolimus and mycophenolate mofetil (MMF) for induction and maintenance therapy in LN (n=40).^18^

All patients were required to have biopsy-proven active LN class III, IV or V according to the International Society of Nephrology/Renal Pathology Society (ISN/RPS) 2003 within 6 months before enrolment. The major exclusion criteria were renal impairment (estimated glomerular filtration rate [eGFR] <25 mL/min/1.73 m^2^), severe extrarenal organ involvement, uncontrolled infection, cytopenia and pregnancy. Those who have completed a 6-month follow-up and have two out of three urine samples collected at month 0 (before induction treatment), month 3 and month 6 for uTWEAK level measurement were enrolled into the study.

### Study protocol

The patients were seen regularly every month. The following routine laboratory evaluations were collected: complete blood count, serum chemistry, serum C3, C4 and anti-dsDNA titres, urinalysis, spot urine protein/creatinine ratio and/or a 24-hour urinary protein. Systemic lupus activity was scored with the original Systemic Lupus Erythematosus Disease Activity Index (SLEDAI) 2000. Histopathological information such as ISN/RPS 2003 classification, crescent formation, activity index (AI) and chronicity index (CI) were extracted from the renal pathology reports.

### Immunosuppressive treatment

Patients received one of the following induction regimens according to the allocated treatment arm (CONTROL and TTT study) or physicians’ discretion (CU cohort): (1) tacrolimus (TTT cohort) was titrated to achieve trough blood concentrations of 6–10 ng/mL in the first 2 months and then 4–8 ng/mL thereafter; (2) MMF (CU, CONTROL and TTT cohort) was given at a dose of 1500–2000 mg/day; (3) EC-MPS (CONTROL cohort) was prescribed at a dose of 1440 mg/day; (4) intravenous cyclophosphamide (CU and CONTROL cohort) was given 0.5–1 g/m^2^ monthly for 6 months; and (5) azathioprine (CU cohort) was given at 1–1.5 mg/kg/day. All patients received concomitant prednisone at a dose of 0.5–0.7 mg/kg/day (maximum 60 mg/day), with tapering by 5–10 mg/day every 2 weeks until a dose of 5 mg/day had been reached, and this dosage was maintained until the end of 24 weeks.

### Primary outcomes

A complete response was defined as normal or ≤25% decline of Modification of Diet in Renal Disease glomerular filtration rate (MDRD-GFR) from baseline and a proteinuria of less than 0.5 g/day. A partial response was defined as normal or ≤25% decline of MDRD-GFR from baseline and at least 50% reduction of proteinuria, with a level more than 0.5–3.0 g/day. The participants who did not meet the above criteria were reported as non-responders.^19^

### Quantification of uTWEAK

At the time of the visit, each patient provided a freshly voided urine specimen which was immediately centrifuged to remove the sediment and kept frozen at −80°C until the test was performed. When it was time to perform the test, the samples were thawed at room temperature. uTWEAK levels were measured in duplicates by human TWEAK DuoSet ELISA kits (R&D Systems, Minneapolis, MN, USA). The tested protocol followed the manufacturer’s manual. Mouse anti-human TWEAK was used as the capture antibody. Biotinylated goat anti-human TWEAK was used as the detection antibody. The standard curve was generated using 7.8–500 pg/mL recombinant human TWEAK. TWEAK levels were corrected to urine creatinine and the levels are therefore expressed as pictograms per milligram of creatinine (pg/mgCr).

### Statistical analysis

Continuous variables were expressed as mean (95% CI). Differences between two groups were analysed by Student’s t-test. Multiple groups were compared using one-way analysis of variance (ANOVA) or repeated measures ANOVA, followed by Bonferroni post hoc test. Associations were tested by linear regression.

A receiver operating characteristic (ROC) curve analysis of the uTWEAK levels was used to compare the ability of the various biomarkers to predict therapeutic response at 6 months. Sensitivity and specificity were derived from the ROC curves and were used to identify the cut-off point for uTWEAK level.

Univariate and multivariate logistic regression was performed to determine the association of uTWEAK and therapeutic response at 6 months. The ORs were obtained from the univariate and multivariate logistic regression models. P<0.05 was considered statistically significant. Statistical analysis was performed using JMP software V.13.2.1 (SAS Institute, Cary, North Carolina, USA) and dot-plot graphs were created using GraphPad Prism V.4.03 (GraphPad Software, La Jolla, CA, USA).

## Results

The demographic characteristics of the patients are presented in table 1 (see online supplementary file 3). A total of 110 patients were included in this study; 29% had complete response to treatment, 34% had partial response to treatment and 37% did not respond to treatment after 6 months of induction therapy. There were 76 renal pathology reports of patients seen at Chulalongkorn University Hospital available for review (69%). Most of the patients were classified as LN class IV (71%). Although not statistically significant, complete responders (CR) tended to have lower AI and CI (mean AI: 6.3 in CR vs 10.4 in partial responders [PR] and 10.5 in non-responders [NR], and mean CI: 1.9 in CR vs 2.2 in PR and 3.4 in NR, respectively). A multigroup comparison between CR, PR and NR groups yielded an overall significant difference in uTWEAK levels and urine protein creatinine index (UPCI) (p=0.046 and 0.02, respectively). Post hoc testing with Bonferroni corrections showed no difference in uTWEAK levels between CR, PR and NR. However, UPCI in the CR group was significantly lower than the PR group (p=0.0001).

10.1136/lupus-2018-000298.supp1 Supplementary data

**Table 1:** Demographic characteristics of the study population

	Complete responders	Partial responders	Non-responders
Patients, n (%)	32 (29)	37 (34)	41 (37)
Age (years)	32 (28–35)	35 (29–38)	31 (29–34)
Sex (female/male)	31/1	35/2	38/3
Immunosuppressive agents (n)			
Cyclophosphamide	6	8	11
Mycophenolate mofetil	18	11	14
Mycophenolate sodium	1	12	8
Tacrolimus	6	6	7
Azathioprine	1	0	1
International Society of Nephrology/Renal Pathology Society (ISN/RPS) 2003 classification (n=76)			
III or III+V	4	1	5
IV or IV+V	15	20	19
V	3	4	5
Activity index (n=40)	6.3 (3.6–9.1)	10.4 (6.8–14.1)	10.6 (3.6–9.1)
Chronicity index (n=40)	1.9 (0.8–3.0)	2.2 (0.7–3.7)	3.4 (2.3–4.5)
Urinary protein to creatinine ratio (g/g)	3.3 (2.5–4.2)	6.2 (4.8–7.7)	5.5 (3.8–7.2)
Serum creatine (mg/dL)	0.9 (0.78–0.99)	1.0 (0.84–1.08)	0.9 (0.78–0.99)
C3 (mg/dL)	83 (41–448)	95 (35–773)	83 (50–112)
Anti-dsDNA (IU)	335 (151–518)	461 (255–668)	484 (0–1013
Serum albumin (g/dL)	2.8 (2.5–3.2)	2.9 (2.6–3.2)	2.7 (2.4–3.0)
uTWEAK (pg/mgCr)	0.58 (0.36–0.81)	2.18 (0.78–3.58)	1.11 (0.65–1.58)

Continuous variables presented as the mean (95% CI).

uTWEAK, urine TWEAK.

### Relationships between uTWEAK and clinical and pathological severity of LN

There was no correlation between uTWEAK level and the magnitude of proteinuria, serum creatinine, serum albumin, C3 level, anti-dsDNA and SLEDAI score at time of flare (data not shown).

Patients were divided into proliferative (ISN/RPS 2003 class 3 and 4) and non-proliferative (ISN/RPS 2003 class 5) groups based on the renal biopsy reports. uTWEAK levels did not differ between both groups (p=0.5) (online supplementary figure 1). There were four patients who had more than 25% crescent. Their uTWEAK levels at time of the flares were similar to those who had crescent less than 25% or none (p=0.3). There was no association between AI or CI and uTWEAK level (p=0.1 and 0.4, respectively).

10.1136/lupus-2018-000298.supp2 Supplementary data

### Serial uTWEAK levels and disease activity

**Figure 1:**
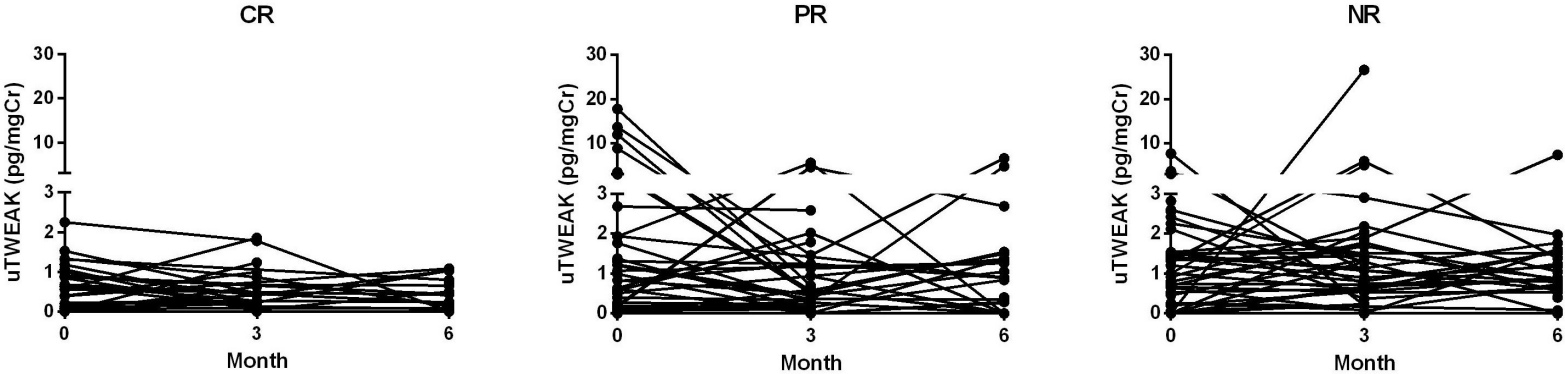
Time course of urine TWEAK (uTWEAK) levels for complete responders (CR), partial responders (PR) and non-responders (NR).

**Figure 2:**
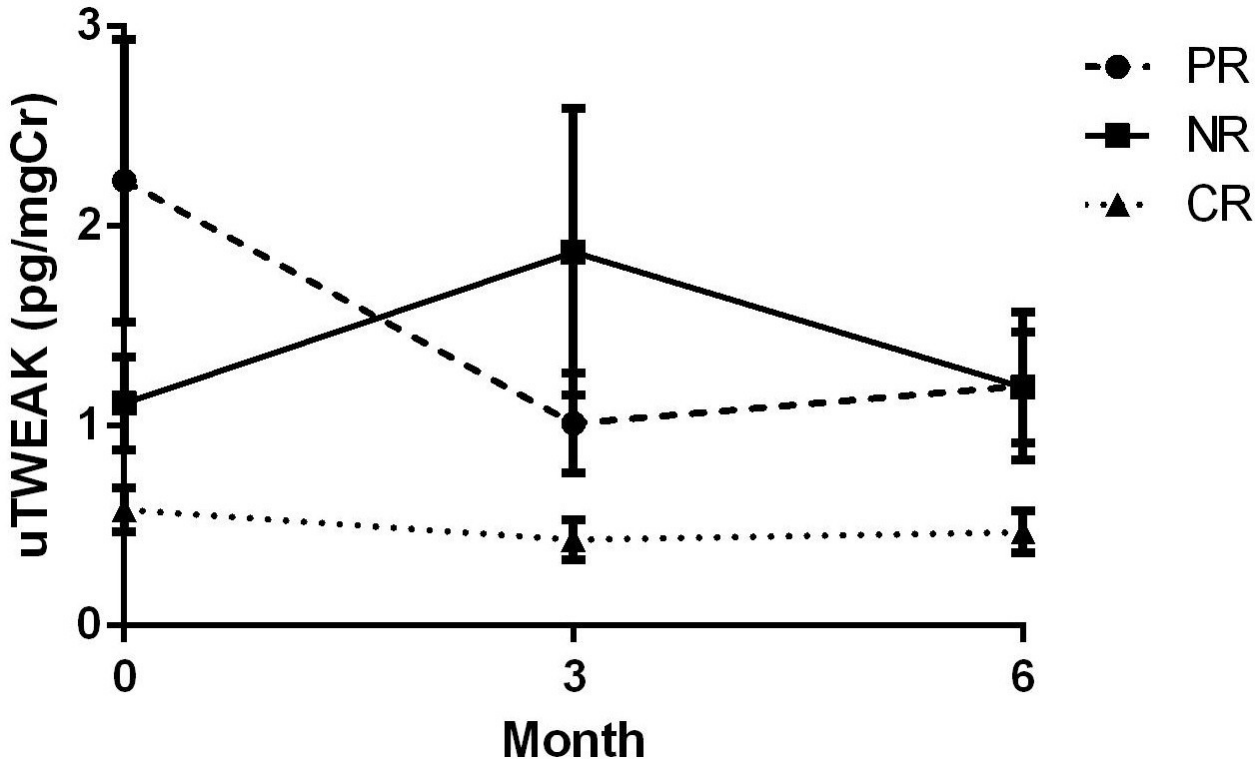
The trends of urine TWEAK (uTWEAK) for complete responders (CR), partial responders (PR) and non-responders (NR). Graph represents means with SE.

A total of 262 samples of uTWEAK levels at different time points were available during the follow-up period (figure 1). Although the changes of uTWEAK levels overtime did not reach statistical significance, different patterns of uTWEAK trends between CR, PR and NR groups were observed (figure 2). On average, NR exhibited a sustained elevation of uTWEAK levels over time, while uTWEAK levels for PR trended down at 3 months, and CR had the least amount of uTWEAK throughout the course of the induction treatment.

The effects of immunosuppressive therapy on uTWEAK were examined. There was no relationship between uTWEAK levels and the cumulative amount of prednisolone received during the preceding 90 days (p=0.9).

### Performance of uTWEAK in predicting renal response at 6 months

After the logistic regression analyses were done, ROC was constructed to assess whether uTWEAK at 0 and 3 months may be used to predict treatment response at 6 months compared with the conventional biomarkers. To predict non-response to 6-month treatment, UPCI and C3 at 3 months were useful (online supplementary table 1A,B). On the other hand, uTWEAK levels and UPCI at 3 months were found to be good predictors for complete response (table 2). In the multivariate analysis, after having adjusted for age, sex and eGFR and type of induction treatment, the OR was 0.39 (95% CI 0.17 to 0.92) for high uTWEAK levels at month 3 which correlated with complete response. However, uTWEAK levels by itself at month 3 were inferior in distinguishing between CR and non-CR compared with UPCI (area under the ROC curve [ROC-AUC] 0.68 vs 0.81, respectively). When uTWEAK was combined with UPCI at month 3, predictive performance for complete response improved (ROC-AUC 0.83, p<0.001).

**Table 2:** The area under a receiver operating characteristic curve of urine TWEAK and clinically used biomarkers to predict complete response at 6 months of therapy

	Month 0		Month 3	
	ROC-AUC	P value	ROC-AUC	P value
uTWEAK (pg/mgCr)	0.6	0.09	0.68	0.02
UPCI (g/g)	0.67	0.01	0.81	0.0006
Creatinine (mg/dL)	0.47	1.0	0.56	0.3
Albumin (g/dL)	0.56	0.7	0.64	0.2
C3 (mg/dL)	0.55	0.3	0.54	0.7

ROC-AUC, area under the receiver operating characteristic curve; TWEAK, TNF-like weak inducer of apoptosis; UPCI, urine protein creatinine index; uTWEAK, urine TWEAK.

Using the ROC curve data and respective specificity and sensitivity values, optimal threshold for uTWEAK level at month 3 was 0.46 pg/mgCr; this level can predict complete response and had a 70% sensitivity and 63% specificity. Table 3 shows the predictive performance of uTWEAK and UPCI.

**Table 3:** Performance of uTWEAK and UPCI in predicting the complete response at 6 months

Variable	Cut-off	Sensitivity (%)	Specificity (%)	Positive predictive value (%)	Negative predictive value (%)	Positive likelihood ratio	Negative likelihood ratio
uTWEAK month 3 (pg/mgCr)	0.46	70	63	42	85	1.89	0.48
UPCI month 0 (g/g)	4.3	78	46	41	86	1.44	0.48
UPCI month 3 (g/g)	1	70	75	53	86	2.80	0.40

UPCI, urine protein creatinine index; uTWEAK, urinary TWEAK.

## Discussion

Prior studies have demonstrated the potential value of uTWEAK as a biomarker for LN. uTWEAK levels were reported to be elevated at flares and higher in SLE with LN than those without LN. In this study, we examined the role of serial measurements of uTWEAK as an early predictor for treatment response among 110 patients with biopsy-proven class III/IV/V LN. At the third month of treatment, a low level of uTWEAK could predict complete response at 6 months. Overall, uTWEAK levels were consistently low in CR, whereas for PR, uTWEAK levels trended down at 3 months and for NR, the uTWEAK levels were persistently elevated. This may reflect the different degree of ongoing renal inflammation which may further guide the treatment plan.

TWEAK plays a major role in renal inflammation through actions on intrinsic renal cells.^20^ The TWEAK levels and expression of its sole receptor Fn14 are relatively low in normal kidney tissues whereas in patients with LN, they are highly expressed in glomerular and tubulointerstitial cells.^21^ TWEAK stimulates the mesangial cells which results in an increase in proinflammatory cytokines such as monocyte chemotactic protein-1, regulated on activation, normal T cell expressed and secreted, and interferon-induced protein-10 in a dose-dependent fashion.^10^ Moreover, Fn14-knockout lupus mice had significantly lower levels of proteinuria, more attenuated glomerular and tubulointerstitial inflammation, significant reduction in glomerular Ig deposition and substantial preservation of podocytes compared with wild-type lupus mice.^22^ All of this evidence suggests that TWEAK is involved in the pathological processes that occur locally in the kidneys of LN. However, a recent phase 2, randomised controlled study of an anti-TWEAK antibody as an adjunctive therapy for active LN has been prematurely terminated as it failed to show benefit, although dose-dependent reductions of serum and urinary TWEAK were observed. The lack of effect could be due to late initiation of anti-TWEAK as the study included only patients with persistent proteinuria after 12 weeks of standard induction therapy. In addition, the antifibrotic effect of TWEAK inhibition demonstrated in animal models remains to be proven with longer follow-up duration in patients with LN.

Current biomarkers for LN such as proteinuria, serum creatinine, anti-dsDNA and complement levels lack sensitivity and specificity to detect renal disease activity in LN. Although proteinuria is the main criteria for response to treatment, it may be from fibrosis/scarring. Persistent proteinuria does not always indicate active inflammation. On the other hand, subclinical renal damage commonly occurs before there is an increase in the serum creatinine or proteinuria.^23^ Complements can also decrease in other immune-complex-mediated lesions, such as vasculitis despite no renal disease activity. Therefore, early sensitive biomarkers reflecting intrarenal inflammation are needed. Our findings revealed that uTWEAK had different trends between CR, PR and NR groups, which are likely to reflect the remnant of inflammation with respect to treatment. CR had relatively low uTWEAK levels, which probably correlated with less active disease as evidenced by numerically lower histological AI (mean: 6.3 [CR] vs 10.4 [PR] and 10.5 [NR]) and less proteinuria at flares (3.3±2.4 [CR] vs 6.2±4.4 g/g [PR] and 5.5±5.4 g/g [NR]). Average downward trend of uTWEAK at 3 months among PR may also indicate early response to treatment whereas for NR, the uTWEAK levels did not change. The result of this study suggested that biomarker monitoring following initial therapy could predict treatment response. Furthermore, early responsiveness to therapy likely leads to good outcome regardless of disease severity at baseline. This highlights the importance of serial biomarker monitoring at the early course of therapy so that the immunosuppression can be modified to achieve the best outcomes.

Although uTWEAK levels may correlate with ongoing renal inflammation, there were some overlaps between responders and non-responders. As expected, the performance of uTWEAK alone to predict renal response was inferior to UPCI, since UPCI itself is a main criterion for treatment response. Notably, NR in this study had numerically higher CI compared with responders (table 1). Persistent proteinuria could be, therefore, from chronic renal damage in some patients. We speculate that the downward trend of uTWEAK levels observed in some NR was due to improvement of the renal inflammation. However, one of the limitations of this study is that the renal biopsy was not repeated after therapy and the data on long-term outcomes of these patients were limited. Follow-up renal biopsy may be considered in future studies to accurately determine renal disease activity. Another limitation is there were multiple induction regimens used although the direct effect of individual induction therapy on TWEAK signalling is unknown. It has been shown in animal model that calcineurin inhibitor upregulated TWEAK receptor expression in renal epithelial cell. It is difficult to determine whether uTWEAK levels were interfered by calcineurin inhibitor even though uTWEAK level at 3 months remained a significant predictor of CR at 6 months even adjusted for type of induction regimen.

Our findings concur with the prior studies that uTWEAK could not distinguish between proliferative LN from membranous LN and had no correlation with renal pathological severity.^13^ Consistent with our findings, a study of glomerular mRNA expression of TWEAK among 42 patients with LN showed that TWEAK was also highly expressed in pure membranous LN.^21^ Thus, currently, there is no evidence to support the role of uTWEAK to predict renal pathology.

The advantage of this study is that it is a relatively large prospective study with serial measurements of uTWEAK levels to forecast outcomes. Although there is no clear cut-off level, the uTWEAK levels combined with other biomarkers should enhance their accuracy. As SLE is a heterogeneous disease, perhaps certain group of patients with LN may benefit from uTWEAK level monitoring and anti-TWEAK therapy. This study has complemented prior works in the search for ‘ideal’ biomarker for LN. In the future, urine biomarkers, including uTWEAK, have the potential to be successfully used to personalise therapy for SLE.

10.1136/lupus-2018-000298.supp3 Supplementary data
