# Supplementary material for: Urine TWEAK level as a biomarker for early response to treatment in active lupus nephritis: a prospective multicentre study
Source: Lupus Sci Med. 2019 Apr 9;6(1):e000298. doi: 10.1136/lupus-2018-000298 (PMC6519400; doi:10.1136/lupus-2018-000298)
Supplement: Supplementary data [file lupus-2018-000298supp001.docx]

|  | **Cohort name** | | |
| --- | --- | --- | --- |
|  | **CU** | **CONTROL** | **TTT** |
| Number of patients (N, %) | 29 (26%) | 39 (35%) | 42 (38%) |
| Age (years) | 31 (27-35) | 33 (30-36) | 32 (29-36) |
| Sex (female/male) | 26/3 | 38/1 | 40/2 |
| Immunosuppressive agents (N, %) |  |  |  |
| Cyclophosphamide | 7 | 18 | 0 |
| Mycophenolate Mofetil or Mycophenolate Sodium | 20 | 21 | 23 |
| Tacrolimus | 0 | 0 | 19 |
| Azathioprine | 2 | 0 | 0 |
| Renal Pathology Society (ISN/RPS) 2003 classification (n=76) |  |  |  |
| III or III+V | 2 | 4 | 4 |
| IV or IV+V | 11 | 13 | 30 |
| V | 1 | 2 | 9 |
| Activity index (n=40) | 9.4 (6.5-12.2) | 10.3 (6.8-13.8) | 9.3 (5.3-13.2) |
| Chronicity index (n=40) | 2.9 (1.4-4.3) | 2.8 (1.4-4.2) | 2.3 (1.1-3.6) |
| Urinary protein to creatinine ratio (g/g) | 3.5 (2.7-4.4) | 7.9 (6.1-9.7) | 3.6 (2.7-4.6) |
| Serum creatinine (mg/dL) | 1.04 (0.90-1.17) | 0.92 (0.80-1.03) | 0.85 (0.77-0.93) |
| MDRD eGFR (mL/min/1.73m2) | 85 (73-100) | 91 (82-101) | 97 (90-105) |
| CKD stage |  |  |  |
| 1 | 13 (45%) | 23 (59%) | 26 (62%) |
| 2 | 9 (31%) | 7 (18%) | 13 (31%) |
| 3 | 5 (17%) | 9 (23%) | 3 (7%) |
| 4 | 2 (7%) | 0 (0%) | 0 (0%) |
| 5 | 0 (0%) | 0 (0%) | 0 (0%) |
| uTWEAK(pg/mgCr) | 1.66 (0.27-3.06) | 1.42 (0.92-1.92) | 1.10 (0.09-2.11) |

**Supplemental table 2. Demographic characteristics of the study population** **by study cohort**

Continuous variables presented as the mean (95% CI)
