# Supplementary figures and images for: Urine TWEAK level as a biomarker for early response to treatment in active lupus nephritis: a prospective multicentre study
Source: Lupus Sci Med. 2019 Apr 9;6(1):e000298. doi: 10.1136/lupus-2018-000298 (PMC6519400; doi:10.1136/lupus-2018-000298)

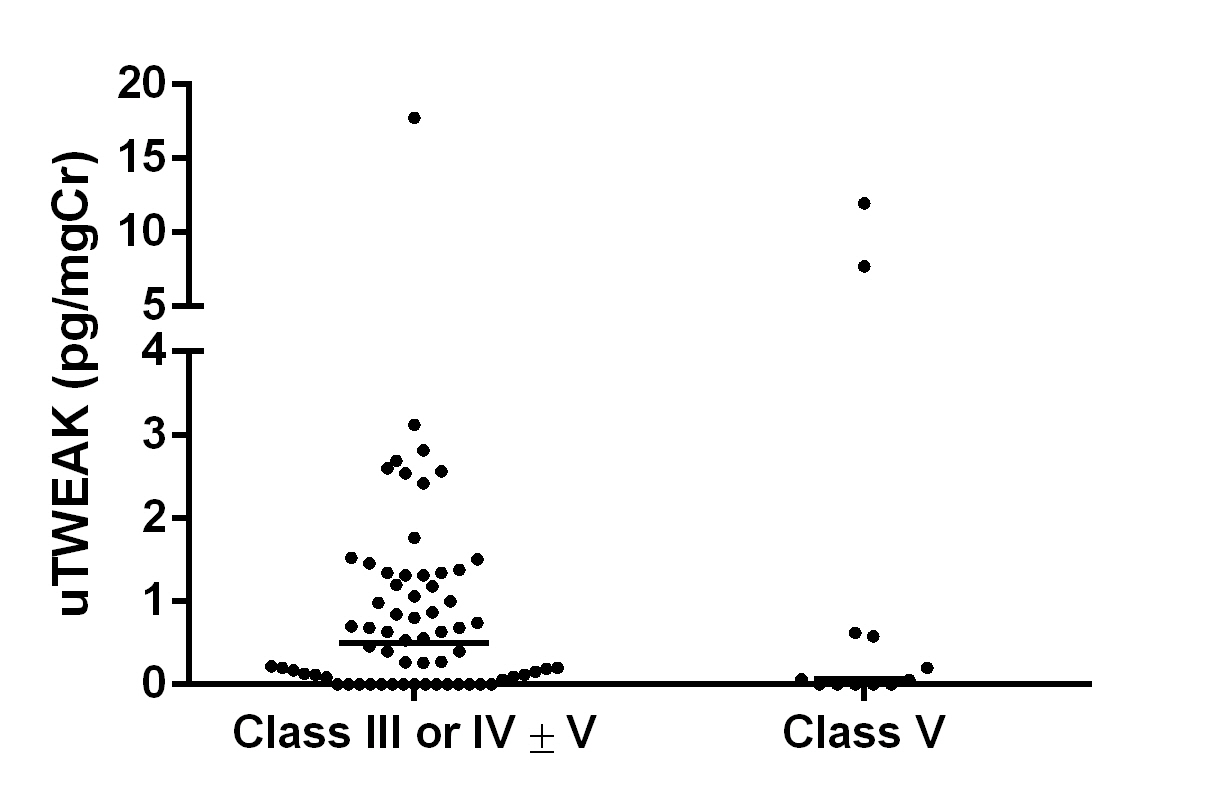

Supplement: Supplementary data [file lupus-2018-000298supp002.jpg]
