# Supplementary material for: Urine TWEAK level as a biomarker for early response to treatment in active lupus nephritis: a prospective multicentre study
Source: Lupus Sci Med. 2019 Apr 9;6(1):e000298. doi: 10.1136/lupus-2018-000298 (PMC6519400; doi:10.1136/lupus-2018-000298)
Supplement: Supplementary data [file lupus-2018-000298supp003.docx]

**Supplemental table 1A.** The area under a receiver operating characteristic curve (ROC-AUC) of urine TWEAK and clinically used biomarkers to predict nonresponse to therapy at 6 months

|  | Month 0 | | Month 3 | |
| --- | --- | --- | --- | --- |
|  | ROC-AUC | p-value | ROC-AUC | p-value |
| uTWEAK (pg/mgCr) | 0.47 | 0.5 | 0.67 | 0.1 |
| UPCI (g/g) | 0.50 | 0.5 | 0.75 | 0.0001* |
| Creatinine (mg/dL) | 0.53 | 0.4 | 0.53 | 0.9 |
| Albumin (g/dL) | 0.57 | 0.3 | 0.67 | 0.1 |
| C3 (mg/dL) | 0.56 | 0.3 | 0.67 | 0.03* |

**Supplemental table 1B.** Performance of UPCI and C3 in predicting nonresponse to therapy at 6 months

| Variable | Cut-off | Sensitivity | Specificity | Positive predictive value | Negative predictive value | Positive likelihood ration | Negative likelihood ratio |
| --- | --- | --- | --- | --- | --- | --- | --- |
| UPCI month 3 (g/g) | 3.0 | 85% | 55% | 75% | 69% | 1.89 | 0.27 |
| C3 month 3 (mg/dL) | 78 | 77% | 54% | 73% | 59% | 1.67 | 0.43 |
